# Flowering time: From physiology, through genetics to mechanism

**DOI:** 10.1093/plphys/kiae109

**Published:** 2024-02-28

**Authors:** Robert Maple, Pan Zhu, Jo Hepworth, Jia-Wei Wang, Caroline Dean

**Affiliations:** Department of Cell and Developmental Biology, John Innes Centre, Norwich Research Park, Norwich, NR4 7UH, UK; Department of Cell and Developmental Biology, John Innes Centre, Norwich Research Park, Norwich, NR4 7UH, UK; Department of Biosciences, Durham University, Stockton Road, Durham, DH1 3LE, UK; National Key Laboratory of Plant Molecular Genetics (NKLPMG), CAS Center for Excellence in Molecular Plant Sciences (CEMPS), Institute of Plant Physiology and Ecology (SIPPE), Chinese Academy of Sciences (CAS), Shanghai 200032, China; School of Life Science and Technology, Shanghai Tech University, Shanghai 201210, China; New Cornerstone Science Laboratory, Shanghai 200032, China; Department of Cell and Developmental Biology, John Innes Centre, Norwich Research Park, Norwich, NR4 7UH, UK

## Abstract

Plant species have evolved different requirements for environmental/endogenous cues to induce flowering. Originally, these varying requirements were thought to reflect the action of different molecular mechanisms. Thinking changed when genetic and molecular analysis in *Arabidopsis thaliana* revealed that a network of environmental and endogenous signaling input pathways converge to regulate a common set of “floral pathway integrators.” Variation in the predominance of the different input pathways within a network can generate the diversity of requirements observed in different species. Many genes identified by flowering time mutants were found to encode general developmental and gene regulators, with their targets having a specific flowering function. Studies of natural variation in flowering were more successful at identifying genes acting as nodes in the network central to adaptation and domestication. Attention has now turned to mechanistic dissection of flowering time gene function and how that has changed during adaptation. This will inform breeding strategies for climate-proof crops and help define which genes act as critical flowering nodes in many other species.

## Introduction

Flowering is a major developmental transition in the life cycle of a plant. Correct timing of this process has a huge impact on reproductive success and has thus been of central importance in plant breeding. Different strategies have evolved to ensure correct timing for successful outcrossing, alignment of flowering with pollinators, and sexual reproduction in favorable external conditions (reviewed in Lee et al. 2023). However, different critical factors influence the timing of the transition when environmental conditions change and flowering is a last defense against acute heat/drought stress, as seeds are more likely to survive (reviewed in Takeno 2016).

Early studies focused on flowering physiology—production of transmissible signals from the leaf to the apex, and changes in reproductive competence of the meristem (reviewed in Bernier et al. 1993). There were 3 major theories for flowering control: the “florigen/antiflorigen” concept (Lang 1984), envisioning a specific floral promoter and inhibitor; the “nutrient diversion” hypothesis (Sachs and Hackett 1983), where modification of source/sink relationships in inductive conditions resulted in the shoot apex receiving a better supply of assimilates; and finally, the “multifactorial control” theory (Bernier et al. 1981; Bernier 1988) postulated that multiple signals involving chemicals, assimilates, and phytohormones synergize to induce the floral transition. These early studies still make good reading and suggest many new molecular experiments.

The *Arabidopsis* genetic revolution then transformed thinking: *Arabidopsis thaliana* mutants (Koornneef et al. 1991; Koornneef et al. 1998) with altered flowering time revealed an integrated network of environmental and endogenous input pathways. These converge to quantitatively control the expression of floral pathway integrators, which when expressed above a certain threshold trigger the transition to flowering (Koornneef et al. 1991; Chandler et al. 1996; Koornneef et al. 1998). This then provided the conceptual framework to explain the diversity of physiological requirements in different species. Although much of our understanding of flowering time has come from studies on *A. thaliana*, significant progress has also been made in other species such as rice, wheat, and the model temperate grass *Brachypodium* (Higgins et al. 2010; Tsuji et al. 2013; Cao et al. 2021). We refer the reader to the following reviews (Osnato et al. 2021; Quiroz et al. 2021; Li et al. 2022a) that cover the extensive recent literature on flowering time control. In this short review we focus on how the molecular understanding of environmental signal integration fits into thinking from earlier physiological analyses. We also discuss how current knowledge may facilitate breeding in crops and pose questions for future research.

**ADVANCES**

- Forward genetics screens in *Arabidopsis thaliana* identified many flowering time mutants and established molecular understanding of the genetic pathways mediating the floral transition.
- Multiple pathways converge on a common set of genes specifying the floral meristem identity, so multiple environmental and endogenous cues can be integrated into the floral transition.
- Natural selection has targeted different nodes of the floral network to adapt flowering time to new environments. Nature has done the mutagenesis for us.
- These have also been selected in the domestication of crop species, although in each species a different node or pathway has been predominantly selected and researched.
- Combining field studies with more accurate simulation in the laboratory is key to understanding flowering time control in natural environments.

## *Arabidopsis*: the Rosetta stone?

**Figure 1.**
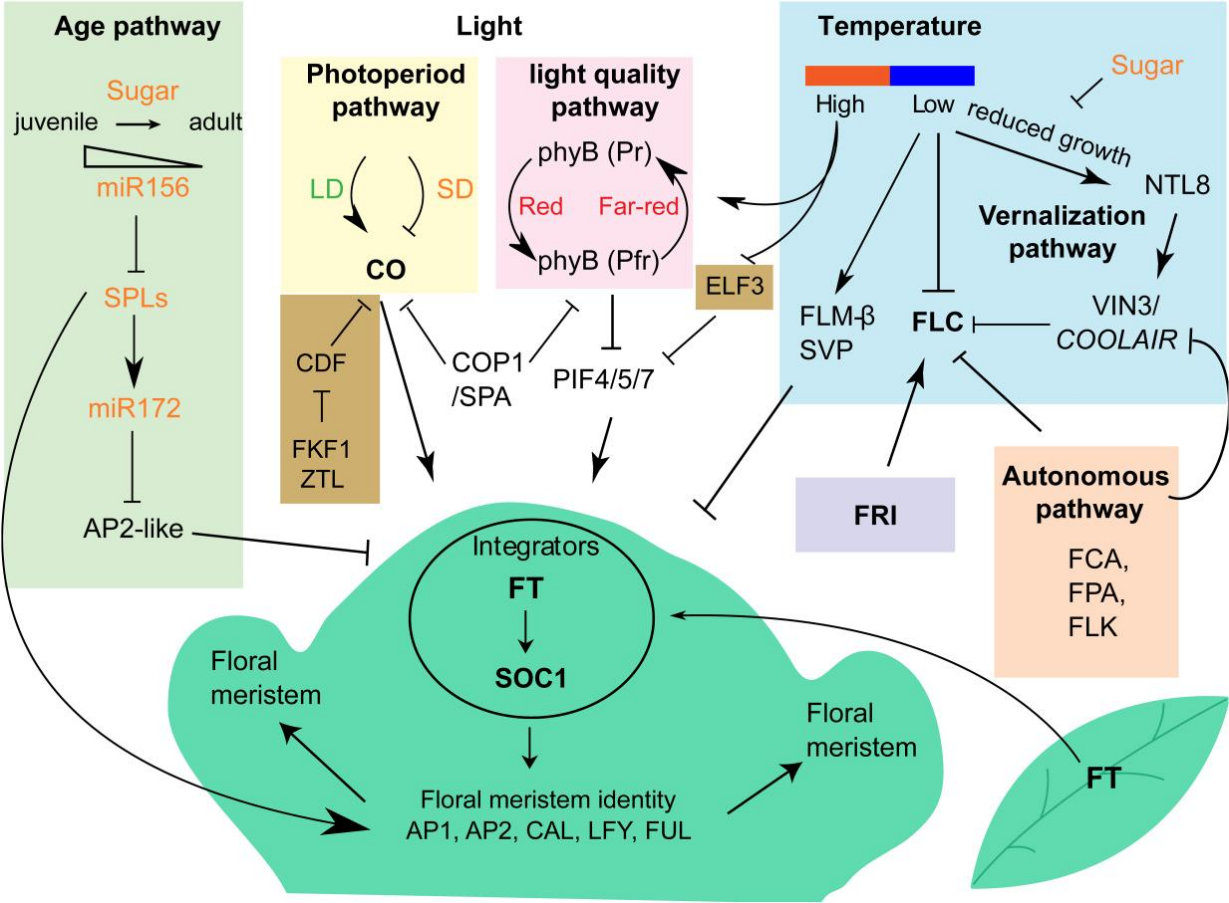
The main genetic pathways controlling flowering time in *Arabidopsis*. Colored boxes highlight different pathways; FRI (purple) and clock components (brown), key integration nodes (FLC, CO, FT, and SOC1), and those with extensive natural variation (FRIGIDA, FLC, and FT) are in bold. Arrows indicate positive and bars represent negative regulatory relationships. Genetic pathways converge on *FT*, encoding a transmissible signaling molecule transported from the leaves to the SAM. The floral pathway integrators (in a circle) and floral meristem identity genes are shown in the green schematic meristem. The influence of sugar on some pathways is indicated. Different pathways are interconnected, for example, photoperiod and light quality and temperature pathways by COP1/SPA, and circadian and high temperature pathways by ELF3.

How did *Arabidopsis* genetics change thinking on flowering time regulation? Forward genetics analyses in *A. thaliana* identified many flowering time mutants (Koornneef et al. 1991). These mutants were categorized into pathways mediating environmental and endogenous cues that promoted the floral transition: maturity, photoperiod, autonomous, vernalization, light quality, and hormonal pathways (Fig. 1). These pathways converge to regulate a common set of genes known as “floral pathway integrators.” These include FLOWERING LOCUS T (FT) and SUPPRESSOR OF OVEREXPRESSION OF CONSTANS1 (SOC1). These in turn regulate the expression of the floral meristem identity genes such as *APETALA1* (*AP1*), *AP2*, *FRUITFULL* (*FUL*), *CAULIFLOWER* (*CAL*), and *LEAFY* (*LFY*). Over time, more and more genes (over 300) have been added to this complex interconnected network of *Arabidopsis* flowering time regulators (Bouche et al. 2016). Such a regulatory network can explain how flowering time can vary in different conditions, how different pathways are predominant in different seasons, and, together with analysis of shoot maturation, how diversity of reproductive strategies evolve. It also enabled the previous physiological understanding of the floral transition—production of transmissible signals from the leaf to the apex and changes in meristem competence—to be described in genetic terms.

### Transmissible floral-promoting signals

Transmissible signals are key to photoperiodic control of flowering. Systemic signaling mechanisms involving long-range inter-tissue transportation integrate the signals received from different parts of the plant (Lifschitz et al. 2006; Lin et al. 2007; Tamaki et al. 2007). The identification of FT as florigen (Kardailsky et al. 1999; Kobayashi et al. 1999) was a major step forward in our understanding of the macromolecules that move through the phloem together with sugars and hormones to regulate flowering and development (Giaquinta 1983; Corbesier et al. 2007; Molnar et al. 2010; Regnault et al. 2015).

The first photoperiodic regulator, the B-box transcription factor CONSTANS (CO), was cloned (Putterill et al. 1995) and shown to transmit photoperiod information to flowering time control through induction of *FT* in leaves. CO is regulated at the transcriptional and posttranslational level by the circadian clock. *CO* mRNA accumulates during the day, peaking 16 h after dawn during long day (more than 12 h of light [LD]) photoperiods (Suarez-Lopez et al. 2001). In the morning *CO* mRNA levels are repressed by CYCLIN DOF FACTORs (CDFs) and through the day, but FLAVIN BINDING, KELCH REPEAT, F-BOX1 (FKF1), and ZEITLUPE (ZTL) relieve CDF-mediated repression in the evening, permitting mRNA accumulation in the evening (Imaizumi et al. 2005; Song et al. 2014). CO protein is itself targeted at night by CONSTITUTIVE PHOTOMORPHOGENIC1 (COP1) and SUPPRESSOR OF PHYA-105S1 (SPA1) for degradation by the proteosome (Valverde et al. 2004; Laubinger et al. 2006; Jang et al. 2008). This complex regulation culminates in CO accumulating only in LD conditions, when light coincides with the evening, to bind to the promoter of *FT* and activate its transcription. Partly as a result of direct CO regulatory dependence on the circadian clock, the mutations in clock component genes, such as *GIGANTEA* (*GI*), *EARLY FLOWERING3* (*ELF3*), and pseudo response regulators (*PRRs*), also affect flowering time (Hicks et al. 1996; Fowler et al. 1999; Nakamichi et al. 2007). However, many clock components have direct transcriptional outputs affecting flowering through other pathways and themselves integrate temperature and light signals—mechanisms that are still being elucidated (for more detailed reviews, see Harmer et al. 2022; Maeda and Nakamichi 2022; Nakamichi et al. 2022; Oravec and Greenham 2022).

In *A. thaliana*, *FT* expression is activated in the companion cells (CCs) in the leaf, and FT protein is then loaded into the sieve elements (SEs) and transported to the SAM, where it forms a floral activation complex with the bZIP transcription factors (TFs), FD and FD-related proteins, as well as 14-3-3 proteins to induce the floral transition (Martignago et al. 2023). Another phosphatidylethanolamine-binding (PEPB) protein related to FT, called TERMINAL FLOWER1 (TFL1), antagonizes FT's function by competition for chromatin-bound FD at shared target loci (Goretti et al. 2020; Zhu et al. 2020). Many studies have shown that long-distance FT transportation is not just a result of diffusion but is highly controlled. The transport of FT protein from CCs to SEs is mediated by FT-INTERACTING PROTEIN 1 (FTIP1), QUIRKY (QKY), and SYNTAXIN OF PLANTS121 (SYP121). FTIP1 is an endoplasmic reticulum (ER) membrane protein that mediates the movement of FT protein through a continuous ER network running through the intercellular connections known as plasmodesmata between CCs and SEs (Liu et al. 2012). QKY and SYP121 (MCTP-SNARE Complex) coordinately facilitate FT export from CCs to SEs through the endosomal trafficking pathway (Liu et al. 2019). After entering into the phloem stream, the long-distance trafficking of FT from leaves to the SAM is regulated by a heavy metal–associated domain-containing protein, NaKR1 (Zhu et al. 2016). However, little is known about how FT is unloaded post-phloem and transported to the shoot apex (Yoo et al. 2013).

FT expression is also promoted by PHYTOCHROME-INTERACTING FACTOR 4 (PIF4) and its orthologs (PIF5 and PIF7) (Kumar et al. 2012; Galvao et al. 2019). Under optimal ambient temperatures, red light converts photoreceptor phyB to the active (Pfr) state, which leads to the degradation of PIF4/5/7 and CO. Under elevated temperatures (27 °C), the active state is rapidly converted to the inactive (Pr) state to allow the induction of *PIF4*/*CO* and subsequently *FT* (Kumar et al. 2012; Fernandez et al. 2016). Flowering time can also be regulated by phyB through PHYTOCHROME AND FLOWERING TIME1 (PFT1) to regulate *FT* transcription (Cerdan and Chory 2003). In addition to *FT* mRNA expression, FT movement from CC to SE is also temperature sensitive; low temperatures facilitate FT sequestration in the cellular membrane of the companion cell due to its phospholipid-binding properties, thus reducing soluble FT levels and delaying flowering (Liu et al. 2020; Susila et al. 2021). FTIP1, QKY, and SYP121 single mutants show different temperature responses, indicating ambient temperature may influence different steps of FT trafficking (Liu et al. 2020). So, FT not only integrates environmental signals from different branches of the floral network but is itself directly regulated by these cues.

### Reproductive competence

Before the floral transition, the shoot apical meristem (SAM) must first become competent. This competence is thought to be associated with the transition from juvenile to adult vegetative phase; however, this is not true for all species (Poethig 2003; Baurle and Dean 2006; Hyun et al. 2017; Poethig and Fouracre 2024). There are some species that flower without a juvenile to adult vegetative phase change, but in many cases, only the adult plants can respond to diverse environmental cues such as photoperiod or low temperature to flower while some others show varied responses in the juvenile and adult phases (Hyun et al. 2017).

The transition from juvenile to adult phase (reviewed in Poethig and Fouracre 2024) is governed by a decrease in expression of microRNA156 (miR156), which represses the expression of SQUAMOSA PROMOTER BINDING-LIKE (SPL) TFs (Wu and Poethig 2006; Wu et al. 2009; Wang et al. 2009a; Yu et al. 2013; Gao et al. 2022). This decrease defines the length of juvenility; a recent study has revealed that the miR156 decline rate is correlated with developmental age rather than chronological age. Upon seed germination, the onset of cell division in the SAM serves as a trigger for the decline in miR156. Concomitant with cell division, the transcriptional activity of *MIR156C* is gradually attenuated by the deposition of the repressive histone mark trimethylation of lysine 27 of histone 3 (H3K27me3) (Cheng et al. 2021). These results provide a plausible explanation for why the decline in miR156 is unidirectional.

Genetic studies and mis-expression experiments have revealed that miR156 regulates flowering time in both leaf and SAM through distinct mechanisms. In leaves, the miR156/SPL module primarily controls flowering via miR172, which targets 5 AP2-like TFs. *MIR172B* is activated directly by SPL9 (Wu et al. 2009). The 5 AP2-like TFs act as flowering repressors by inhibiting the expression of the florigen gene *FT*, which normally contributes to signaling from leaves to meristem. Overexpression of miR172 leads to early flowering, while the simultaneous mutation of 5 *MIR172* genes results in late flowering, particularly under non-inductive conditions (Aukerman and Sakai 2003; Chen 2004; Lian et al. 2021; Ó’Maoileidigh et al. 2021).

Within the shoot apex itself, miR156-targeted SPLs (mainly SPL15) and FT promote flowering directly by activating a shared set of targets, including *AP1*, *FUL*, *LFY*, and *SOC1* (Wang et al. 2009a; Yamaguchi et al. 2009). Additionally, SPL15 releases the inhibition of flowering by AP2 itself through activating *MIR172A* and *MIR172D* (Lian et al. 2021; Ó’Maoileidigh et al. 2021). These findings demonstrate the highly redundant activities and feed-forward action of the miR156/SPL and FT modules in regulating flowering while also revealing the interplay between meristem competence and photoperiod.

Vernalization removes the floral repressors responsible for reducing the sensitivity of the meristem to inductive signals. In Arabidopsis, the major repressor is *FLOWERING LOCUS C* (*FLC*), whose expression is upregulated by *FRIGIDA* (*FRI*). *FLC*, a MADS-box transcription factor, forms a heterodimer with SHORT VEGETATIVE PHASE (SVP; also a MADS-box TF) to negatively regulate *FT* and *SOC1* and thereby prevent flowering (Michaels and Amasino 1999; Searle et al. 2006). Progressive cold treatment represses *FLC* through a co-transcriptional mechanism involving *FLC* antisense transcripts (known as *COOLAIR*) (see Box 1) and in parallel is epigenetically silenced by Polycomb Repressive Complex 2 (PRC2) with both processes regulated through NTL8 (De Lucia et al. 2008; Csorba et al. 2014; Zhao et al. 2020; Zhao et al. 2021; Nielsen et al. 2024). PRC2 associates with a sense noncoding transcript, *COLDWRAP*, derived from the promoter region of *FLC* (Heo and Sung 2011; Kim et al. 2017), involved in the formation of a repressive intragenic chromatin loop at *FLC* (Kim and Sung 2017). A second sense noncoding, intronic transcript, *COLDAIR*, is also proposed to interact with PRC2 (Heo and Sung 2011), but its identity and function are still not fully resolved. The PRC2-induced epigenetic silencing is mitotically stable when temperatures rise in the spring but is reset every generation (Sheldon et al. 2008; Crevillen et al. 2014). Early forward screens uncovered *VERNALIZATION1* (*VRN1*), *VERNALIZATION2* (*VRN2*), *VERNALIZATION INSENSITIVE3* (*VIN3*), and *VERNALIZATION5* (*VRN5*), all of which compromise the plant's ability to establish or maintain stable *FLC* silencing (Chandler et al. 1996; Greb et al. 2007). During the cold, VIN3 protein accumulates and associates with the nucleation region of *FLC* (Sung and Amasino 2004; Finnegan et al. 2005; Wood et al. 2006), where it functions with the VRN2-PRC2 complex through its interaction with VRN5 (Yang et al. 2017; Franco-Echevarria et al. 2023). PRC2 catalyzes the deposition of H3K27me3 around the nucleation region and when the plant is returned to warm conditions, this modification spreads across the whole locus to silence *FLC*. H3K27me3 is stable through cell division and maintains *FLC* in an epigenetically silenced state (Bastow et al. 2004).

Box 1. **The environmental sensitivity of *FLC* antisense transcription.**

A set of cold-induced antisense transcripts, named *COOLAIR*, is expressed at the *FLC* locus in *Arabidopsis thaliana* (Swiezewski et al. 2009). *COOLAIR* initiates immediately downstream of the major sense *FLC* poly (A) site, can transcribe through to the *FLC* promoter, and plays many roles in *FLC* silencing (Csorba et al. 2014; Kim et al. 2017; Nielsen et al. 2024). *COOLAIR* homologs in the semi-perennial relative *Arabis alpina* are induced each winter (Castaings et al. 2014). *COOLAIR* transcripts adopt multiple secondary structures with different conformational dynamics, influenced by temperature (Hawkes et al. 2016; Yang et al. 2022). Monocot *FLC* homologs also show cold-induced antisense transcripts (Jiao et al. 2019).

Cold temperature not only promotes *COOLAIR* RNA levels but also affects its processing; promoting the use of a proximal polyadenylation site and enhancing splicing to form a distal *COOLAIR* isoform called Class II.ii (Zhao et al. 2021; Zhu et al. 2021) (Box 1 Fig. A). Several cold-responsive TFs facilitate cold induction of *COOLAIR* including NTL8, CRT/DRE-binding factors (CBFs), and the group-III WRKY transcription factor WRKY63 (Zhao et al. 2020; Hung et al. 2022; Jeon et al. 2023) (Box 1 Fig. A). These factors have distinct cold sensitivities; for example, CBFs are upregulated upon short cold (minutes/hours) exposure (Jeon et al., 2023), while NTL8 accumulates over weeks of cold exposure (Zhao et al. 2020). The slow timescale of NTL8 induction is due to an indirect thermosensory mechanism whereby cold slows cell division enabling NTL8 protein accumulation through reduced dilution. Thus, CBFs and NTL8 are likely to be responsible for *COOLAIR* induction at different stages of vernalization. Because components of the growth medium, particularly sugar levels, change plant growth rate and influence protein accumulation, *COOLAIR* expression peaks at different stages when analysed in different laboratories (Box 1 Fig. B).

There are contradictory conclusions on the role of *COOLAIR* in cold-induced *FLC* silencing. These are based on knockdown/out mutants generated using different genetic methods (Zhu et al. 2021; Jeon et al., 2023; Zhang et al. 2023a; Zhu and Dean 2023). None of the antisense mutants entirely remove antisense transcription; when transcription is suppressed from 1 region it initiates in another within the locus (Zhao et al. 2020; Zhu and Dean 2023). In addition, the overlap of the *COOLAIR* transcription start region containing transcription factor binding sites, with the sense transcript *FLC* 3′ UTR end, makes it difficult to completely remove the cold sensitivity of *COOLAIR* (Box 1 Fig. A). The combination of molecular analyses with computational modelling helped explain the contradictory findings. *FLC* is silenced through pathways that function with different dynamics: a *COOLAIR* transcription-mediated pathway capable of fast response; and in parallel a slow Polycomb Repressive Complex 2 (PRC2) switching mechanism that maintains each allele in an epigenetically silenced (Nielsen et al. 2024). The parallel repressive inputs and extensive feedback make the mechanism counter-intuitive but provide great flexibility to the plant to cope with ever-changing seasonal conditions.

**Figure A.**
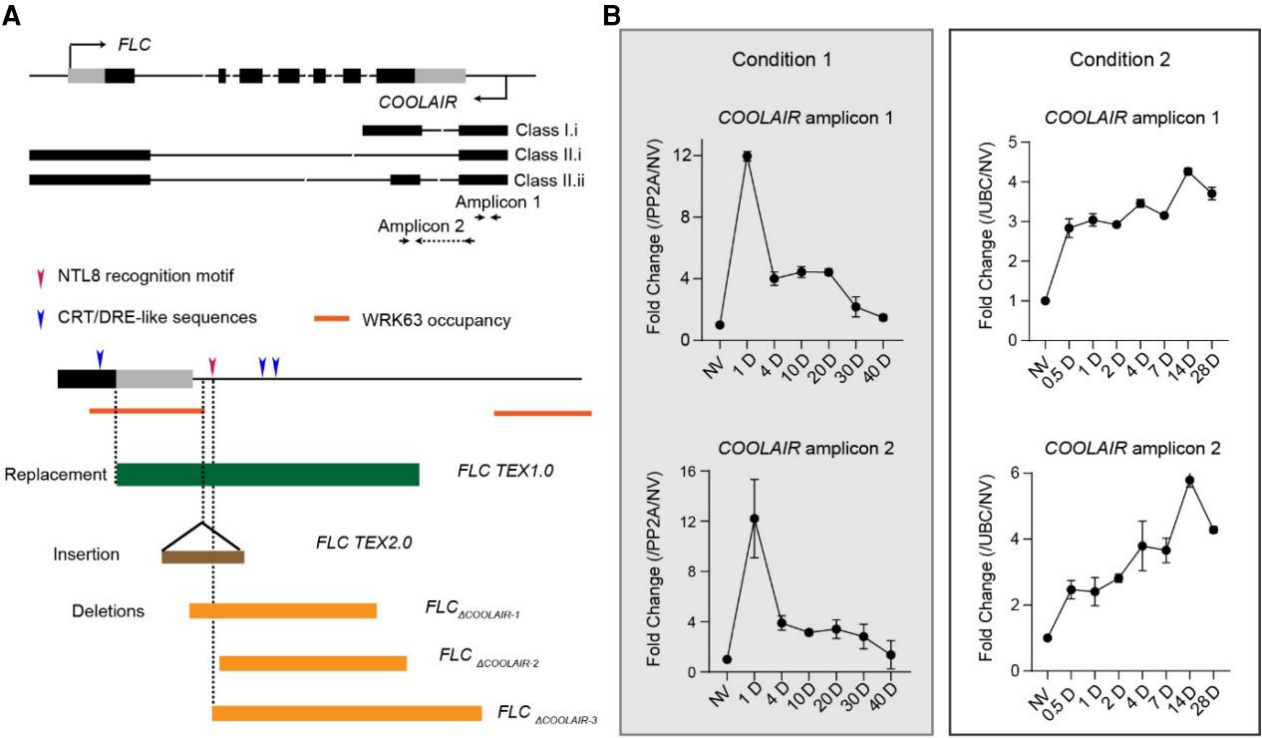
Antisense transcription at *FLC* locus. **A)** Schematic illustration of *FLC* gene architecture and *COOLAIR* transcripts. Black lines represent introns, black boxes represent exons, and grey boxes indicate UTR regions. *FLC* and *COOLAIR* transcription start sites are shown by black arrows. The 3′ end of *FLC* is enlarged below to show binding motifs/regions and currently available mutants that disrupt *COOLAIR*. **B)***COOLAIR* expression profile during cold treatment, measured using Q-RT-PCR and 2 amplicons shown in (A) at different conditions. The data in condition 1 is from Jeon et al., 2023 while the condition 2 is the same as described in Swiezewski et al. 2009.

In addition to *FLC*, other floral repressors play important roles in temperature sensitivity of the Arabidopsis floral transition. FLOWERING LOCUS M (FLM/MADS AFFECTING FLOWERING1), a MADS-box transcription factor related to FLC, represses *FT* and *SOC1* (Balasubramanian et al. 2006a). The *FLM* locus is transcribed into a number of different isoforms via temperature-dependent alternative splicing mechanisms (Pose et al. 2013; Lutz et al. 2015; Sureshkumar et al. 2016). At low ambient temperatures, transcription of the repressive *FLM*-β isoform, which contains the functional DNA binding domain, is promoted compared with other isoforms, allowing FLM to multimerize with SVP and FLC to repress floral integrators (Lee et al. 2013a, 2013b; Pose et al. 2013). At elevated temperatures, active SVP protein is reduced via decreased transcription and increased proteasomal degradation, permitting accelerated flowering (Lee et al. 2007; Lee et al. 2013a, 2013b; Jin et al. 2022). However, mutations in *FLM* and *SVP* have relatively small effects on *A. thaliana* flowering time.

Natural winter annual *A. thaliana* accessions show a *FRI/FLC*-dependent dominant requirement for vernalization, in contrast to the mutation-induced flowering mutants such as *fca* (*flowering locus ca*), *fpa* (*flowering locus pa*), and *flk* (*flowering locus k*) that confer a recessive vernalization requirement—rather like the *Vrn* loci in wheat. These mutants flower late regardless of day length but respond to vernalization or growth in far-red light and were classified into the autonomous floral pathway (Koornneef et al. 1991; Koornneef et al. 1998). The components typically regulate flowering by limiting *FLC* expression levels. FCA, FPA, and FLK are general RNA binding and 3′ processing factors that regulate *FLC* through a transcription-coupled chromatin silencing mechanism (Macknight et al. 1997; Schomburg et al. 2001; Lim et al. 2004; Marquardt et al. 2006). This involves proximal transcription termination linked to delivery of a chromatin environment that affects transcriptional output—initiation, processivity, and elongation (Liu et al. 2007; Baurle and Dean 2008). The proximal termination process influences both *COOLAIR* transcripts from *FLC* and the sense *FLC* transcription (Box 2) (Schon et al. 2021; Menon et al. 2023).

Box 2. **Feedback that complicate analysis of *FLC* and its sensitivity to co-transcriptional regulators.**

The feedback between transcription and chromatin is central to regulation of expression at *FLOWERING LOCUS C* (*FLC*). These mechanisms are difficult to tease apart without support of computational modelling (Menon et al. 2023). Both transcriptional activation by FRIGIDA and repression by the autonomous pathway involve co-transcriptional pathways that link transcription termination with delivery of a changed chromatin environment. This chromatin environment then feeds back to affect transcriptional output by changing transcription initiation, processivity and elongation. FRIGIDA promotes *FLC* transcription by acting as an anti-terminator in the developing embryo, enhancing usage of distal termination sites for both sense and antisense transcription (Schon et al. 2021). The higher transcription delivers active chromatin modifications to the locus (H3K4me, H3K36me3), which enhance distal site usage (Liu et al. 2010). FCA represses *FLC* transcription by promoting proximal termination of both sense and antisense transcription (Menon et al. 2023). This is linked via FLD-mediated H3K4 demethylation to a changed chromatin environment that reduces transcriptional output by feeding back to enhance use of the proximal polyadenylation site (Liu et al. 2007; Liu et al. 2010). These feedback result in counter-intuitive outcomes on steady state RNA levels. For example, loss of FCA primarily reduces the relative propensity for proximal termination, but loss of that step affects the chromatin environment at the whole locus, which results in higher transcription of all the *FLC* and *COOLAIR* transcripts, including proximal *COOLAIR* (Liu et al. 2010). Thus, analyzing changes in absolute levels at steady state can give confusing answers. The answer has been to measure the ratio of proximal to distal polyadenylation. However, sufficient sequencing depth is required to reliably measure these low abundance transcripts. 3′RNA sequencing approaches provide useful data on polyadenylation of both *FLC* and *COOLAIR* (Box 2 Fig. A-B) (Schon et al. 2021; Menon et al. 2023), and these confirm all the analyses using PCR (Liu et al. 2010).

These 3′ sequencing approaches show the extent of mis-regulation in the Arabidopsis genome when functionality of FRI or autonomous pathway components is disrupted. Several hundred genes are differentially transcribed in these genotypes (with significant *P*-values when compared with Col-0), *fca-9* 104 up, 69 down; *fld-4* 201 up,152 down; ColFRI 511 up, 420 down; padj < 0.05. This agrees with their predicted roles as general co-transcriptional regulators. However, it is striking that *FLC* is in each case the most affected gene, with the highest fold change: 10.7 for *fca-9*, 10.8 for *fld-4* and 14.3 for ColFRI (Box 2 Fig. **C)**. What confers this sensitivity to general acting co-transcriptional regulators is a fascinating question, with major implications for why *FLC* has become the node for flowering time variation in the Brassicaceae (Li et al. 2014).

**Figure B.**
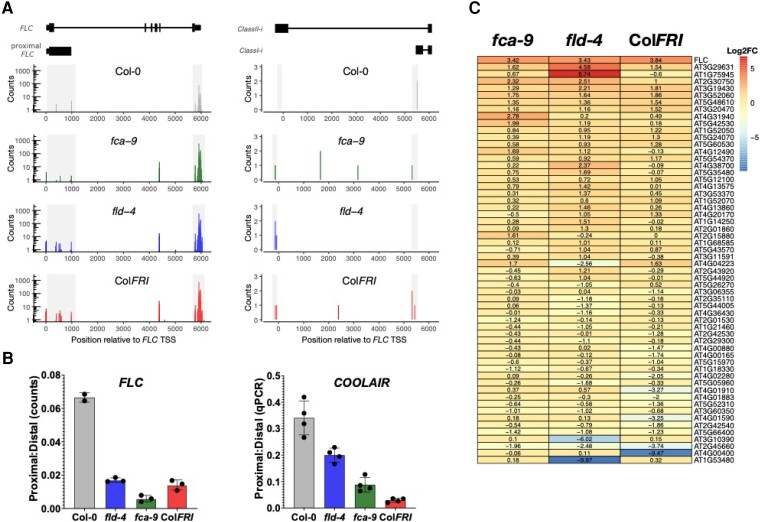
Co-transcriptional 3′processing at the *FLC* locus. **A)** RNA 3′ sequencing at the *FLC* locus reveals altered polyadenylation site selection of both the sense (left) and antisense (right) transcripts by *fca-9*, *fld-4* or Col*FRI* compared with Col-0. **B)** The proximal to distal polyadenylation ratio of *FLC* by 3′RNA sequencing matches conventional qPCR analysis of *COOLAIR* polyadenylation ratio. **C)** Differentially expressed genes in *fca-9*, *fld-4* and Col*FRI* compared with Col-0 (padj < 0.05 and Log2FC > 1). Consistently, *FLC* is one of the top upregulated genes.

## Genes identified through flowering time mutants frequently encode general developmental and gene regulators

The molecular analysis of genes in the Arabidopsis integrated flowering network has led to important mechanistic information as to how organisms can perceive different environmental signals and integrate them over time. The surprise has been that despite having relatively specific flowering time mutant phenotypes, many genes were found to encode general developmental and gene regulators. We discuss a few examples below.

*FT* encodes florigen. FT is a member of the PEBP family that includes *TWIN SISTER OF FT* (*TSF*), *MOTHER OF FT* (*MFT*), and *TFL1* (Yamaguchi et al. 2005; Xi et al. 2010; Hiraoka et al. 2013). In *A. thaliana*, *FT*, *TSF*, and *TFL1* jointly affect shoot architecture through differential activation of axillary meristems, and *FT*, *MFT*, and *TFL1* all affect seed development (Hiraoka et al. 2013; Chen et al. 2014; Zhang et al. 2020a). In other species, the roles for the PEBP family have proliferated: in potato (*Solanum tuberosum*), the *FT*-*like SELF-PRUNING 6A* (*StSP6A*) gene is activated in leaves under short-day conditions (SDs) due to the inactivation of the FT-like repressor *SELF PRUNING 5G* (*StSP5G*) by unstable StCOL1 (Navarro et al. 2011; Abelenda et al. 2016). Along with 2 other SD-activated transmissible FT-like proteins, StSP3D and FT-like 1 (StFTL1), SP6A protein is then transported via phloem from leaves to stolons, where it forms a floral activation-like complex, termed the tuberigen activation complex, which promotes tuber formation (Teo et al. 2017; Jing et al. 2023). A similar shoot-to-root translocation is found in soybean (*Glycine max*), where the shoot-derived ortholog of *Arabidopsis* FT, GmNN1/FT2a, triggers nodulation upon rhizobial infection (Kong et al. 2010; Sun et al. 2011; Li et al. 2022b). In the root, GmNN1/FT2a interacts with GmNFYA-C to activate symbiotic signaling through the GmNFYA-C-ENOD40 module (Li et al. 2022b). Therefore, the mobile signaling functions of FT-like proteins, first established in flowering time control, appear to be involved in many other environmentally controlled development processes.

Other genes first identified through their flowering time phenotype have now also been found to have additional functions. For instance, miR156-targeted SPL TFs influence leaf morphological changes associated with developmental progression (Poethig 2010), male fertility (Xing et al. 2010), nodulation (Wang et al. 2015; Yun et al. 2022), defense against insects (Mao et al. 2017), and anthocyanin biosynthesis (Gou et al. 2011). PFT1 encodes MED25 of the plant Mediator complex and plays an essential role in transcription initiation, regulating jasmonate signaling, biotic and abiotic stress responses, and flowering (Backstrom et al. 2007; Kidd et al. 2009; Inigo et al. 2012).

Many mutants affecting *FLC* expression were found to encode core transcription regulators. These include ELF7 (PAF1) and other Paf1C components, FRI (and FRIc), CAP-BINDING PROTEIN20 (CBP20), and CBP80 (He et al. 2004; Geraldo et al. 2009); splicing regulators: the apoptosis and splicing–associated protein (ASAP) complex and PRE-MRNA PROCESSING8 (PRP8) (Marquardt et al. 2014; Mikulski et al. 2022); RNA binding proteins: THO/TREX, FCA, and FY (Liu et al. 2007; Xu et al. 2021); 3′ processing factors: CPSF, CstF64, CstF77, ANTHESIS PROMOTING FACTOR1 (APRF1), and TYPE ONE SERINE/THREONINE PROTEIN PHOSPHATASE4 (TOPP4) (Liu et al. 2010; Mateo Bonmati et al. 2023). This raises the question: What makes *FLC* such a sensitive target to generic transcriptional regulators? Analysis of fold changes in genes mis-expressed in *fca* reveal the sensitivity of *FLC* regulation compared with other targets (Box 2). Similarly, the vernalization mediators VRN2, VRN5, and VIN3 are PRC2 core and accessory proteins, respectively, and associate widely with loci across the *Arabidopsis* genome (Franco-Echevarria et al. 2023). Redundancy and subfunctionalization between PRC2 forms may explain how some of the mutants have more vernalization-specific phenotypes. However, this relative specificity has provided a nonlethal platform for investigation of the mechanisms of these important general regulators, allowing ongoing insights into conserved regulation of transcriptional processing and epigenetic chromatin memory.

Even the floral repressor *FLC* is not specific to the floral transition. *FLC* is known to bind to many genes and is associated with broader developmental contexts such as seed dormancy, cold tolerance, juvenile-to-adult phase transition, and inflorescence patterning (Chiang et al. 2009; Deng et al. 2011; Huang et al. 2013; Mateos et al. 2017; Auge et al. 2019). Moreover, FRI and FLC increase the stress tolerance of plants during drought and pathogen infection (Wilson et al. 2013; Chen et al. 2018; Chen et al. 2022; Shukla et al. 2022; Xu et al. 2022). The naming of genes based on original forward screens implies a specificity to their function, which in reality is not the case. This convention has caused confusion in the whole field and is not exclusive to the flowering time field.

So, if the genes classified in the regulatory network affecting flowering actually function in many processes important in plant growth and development, which genes are most influential in determining flowering in natural conditions and how have these evolved? The study of natural variation of flowering time has been most helpful in answering these questions.

## Natural variation has identified critical nodes driving flowering time diversity

We summarize studies of the genetic basis of natural variation in flowering, focusing on Arabidopsis and its relatives as 1 example, and then contrast those findings with our understanding of the loci selected during domestication of our major crops.

### Flowering time variation within Arabidopsis and relatives

*Arabidopsis thaliana* accessions show considerable variation for many aspects of flowering time. This has enabled adaptation to their wide climate envelope, from the Arctic Circle to near the equator (Hoffmann 2005). Studies of natural variation of flowering time have focused on vernalization requirement (winter vs rapid-cycling habit), vernalization response, or photoperiod sensitivity. Variation in vernalization requirement was first mapped by Klaus Napp-Zinn in the 1950s, who showed that, despite the quantitative nature of flowering time control, *FRI* could be mapped as a single Mendelian locus (Napp-Zinn 1957; Clarke and Dean 1994). Subsequent QTL analyses between a range of winter annual and rapid cycling accessions showed approximately 70% of winter annual/rapid-cycling variation can be accounted for by allelic variation at *FRI* (Le Corre et al. 2002; Gazzani et al. 2003; Shindo et al. 2005; Werner et al. 2005a; Méndez-Vigo et al. 2011; Strange et al. 2011; Ågrena et al., 2013; Kinmonth-Schultz et al. 2021). Molecular analysis has shown loss-of-function *FRI* mutations are a recurrent feature in the evolution of the rapid-cycling habit (Johanson et al. 2000; Shindo et al. 2005). Two amino acid polymorphisms in the central domain of the FRI protein that change stability or subcellular localization also cause early flowering (Zhang et al. 2020b). *FRI* alleles are associated with flowering time plasticity in regions experiencing high annual temperature variation (Fournier-Level et al. 2022), which may be related to its temperature-sensitive properties (Zhu et al. 2021). The evolutionary relevance of FRI variation extends to *Cardamine hirsuta*, where 3 distinct *FRI* loss-of-function alleles associate with early flowering (Baumgarten et al. 2023).

Variation in vernalization response mapped to *FLC* in QTL analysis of variation between different winter annual types (Shindo et al. 2006). Analysis of the *FLC* genomic sequence in the natural accessions revealed ∼20 major haplotypes, with 5 widely represented in the worldwide population. These haplotypes are distinguished by noncoding SNPs, which were shown to be causative for the different *FLC* expression levels and response to cold (Li et al. 2014). In many cases 1 noncoding SNP has a substantial effect on the phenotype (Li et al. 2014; Li et al. 2015; Qüesta et al. 2020; Zhu et al. 2023). In natural populations these SNPs accumulate over time, driven by natural selection, but their effect may be reduced or enhanced by epistatic interactions in the genetic background (Neto and Hancock 2023). The different causative SNPs at *FLC* are an excellent tool to dissect mechanism and act through influence on promoter activity, epigenetic silencing, or antisense alternative splicing (Li et al. 2015; Qüesta et al. 2020; Zhu et al. 2023). The major haplotypes of *A. thaliana FLC* have been introgressed into a common background to generate a set of near-isogenic lines. Use of these lines in field experiments across Sweden and the UK revealed that autumnal *FLC* expression determined by the noncoding SNPs was the most important determinant modulating flowering time and fitness in response to different natural fluctuating environments (Hepworth et al. 2020).

The variation in *FLC* epigenetic silencing is also a feature in Arabidopsis relatives. In the annual *A. thaliana*, the floral meristem is determinate and growth ceases, but for *Arabis alpina*, a polycarpic perennial, some meristems remain vegetative for growth in the following year. This requires reactivation of the *FLC* ortholog *PERPETUAL FLOWERING1* (*PEP1*) (Wang et al. 2009b). Unlike *FLC* in *Arabidopsis*, *PEP1* repression by cold is not epigenetically stable (Wang et al. 2009b). In *A. alpina*, miR156 repression of *SPL15* in younger meristems prevents conversion of that meristem to flowering post vernalization, even in long photoperiods. *FLC* reactivation is required to prevent high *FT* expression overriding this, as it can in young *A. thaliana* shoots (Hyun et al. 2019). This strategy for perennial growth is not isolated: in *Arabidopsis halleri*, *Arabidopsis lyrata*, *Cardamine hirsute*, *Capsella rubella*, *A. alpina*, and its close annual relative, *Arabis montbretiana*, *FLC* orthologs also influence flowering in an expression-dependent manner and contribute to natural variation and life histories (Wang et al. 2009b; Aikawa et al. 2010; Guo et al. 2012; Kemi et al. 2013; Kiefer et al. 2017). *FLC cis* variation in 3 orthologs include gene duplications and noncoding changes to promoters and introns (Albani et al. 2012; Kemi et al. 2013; Kiefer et al. 2017). Conservation of *COOLAIR* sequences and structures across the Brassicaceae suggests the antisense functionality is also conserved (Castaings et al. 2014; Hawkes et al. 2016; Kiefer et al. 2017).

FT is an important contributor for major-effect QTLs underlying flowering time variation in response to environmental conditions, such as light and temperature (Schwartz et al. 2009; Li et al. 2010; Strange et al. 2011). In *A. thaliana*, long photoperiod induction of *FT* expression is controlled by 2 interdependent regulatory regions, with the distance between them essential for the responsiveness (Schwartz et al. 2009; Adrian et al. 2010). One polymorphism in 1 of the *cis* elements, the CCAAT box (C block), in the Ull2-5 accession is causative for impaired *FT* expression pattern in response to LD induction (Strange et al. 2011). Natural promoter length variation at *FT* creates promoter length differences that correlate with longitudinal and latitudinal clines (Liu et al. 2014). The bHLH transcription factor MYC3 competes with CO to repress the expression of *FT* under unfavorable photoperiods (Bao et al. 2019). The MYC3 binding site, the ACGGAT motif, is specifically present in accessions bearing the long *FT* promoter variant situated toward more northern latitudes (Bao et al. 2019). It should be noted that FT can be functionally converted to TFL1 and vice versa by a single amino acid substitution, and such mutations have been selected during crop domestication (Hanzawa et al. 2005). However, it is still unclear how these mutations are linked to the protein function, especially their movement; TFL1 moves within SAM only, outward from the central region (Conti and Bradley 2007), in a manner very different from FT.

Other genes showing natural variation in both *A. thaliana* and relatives include photoperiod and vernalization regulators. *VIN3* underlies GWAS and QTL peaks in *A. thaliana* and *Brassica napus* (Dittmar et al. 2014; Song et al. 2020). The blue light receptor gene *CRYPTOCHROME2* (*CRY2*) is a rare QTL in *A. thaliana*. In a recent study of the evolution of the ruderal weed species *Cardamine occulta*, CRY2 was selected for photoperiod insensitivity, as well as *FLC* (Li et al. 2023a; Li et al. 2023b). In addition, the photoreceptors *PHYTOCHROME C* (*PHYC*), *PHYB*, and *CO* are likely targets to explain phenotypic variation in other studies (Balasubramanian et al. 2006b; Caicedo et al. 2009; Salome et al. 2011; Rosas et al. 2014). Apart from these pathways, variant alleles at *FLM* have also been associated with flowering time in *A. thaliana* (Lutz et al. 2015; Lutz et al. 2017; Kinmonth-Schultz et al. 2023) as well as *MADS AFFECTING FLOWERING2* (*MAF2*, a relative of *FLM*), *SVP*, *GIS5*, and *HUA2* albeit at lower frequency (El-Din El-Assal et al. 2001; Werner et al. 2005b; Wang et al. 2007; Filiault et al. 2008; Schwartz et al. 2009; Méndez-Vigo et al. 2013; Fournier-Level et al. 2022; Kinmonth-Schultz et al. 2023).

In summary, the natural variation in flowering time predominantly influences either light or temperature pathways. Natural variation for alternative (less predictable) environmental conditions such as light quality, annual rainfall, drought, or heat stress have yet to be fully explored (Kobayashi et al. 2013; Yeoh et al. 2017). To date these have been categorized as stress factors and linked indirectly to flowering time (Takeno 2016).

### Crop domestication

Flowering time variation has been a major trait during crop domestication. The current growing regions of many crop species are frequently very far from their centers of origin. As such, many aspects of their biology have been bred to accommodate the latitudinal range expansion. Traits such as annual, biennial, and perennial habits; ability to grow in high-density monoculture; and shoot architecture have all been modified as breeders selected for higher yield (Gaudinier and Blackman 2020; Liang et al. 2021). Initial domestication likely selected unconsciously for predictability of flowering time and maximizing the growing period to improve yield. Further crop range expansion required local adaptation of the crop to the new environmental conditions and involved selection for variants alleles (Gaudinier and Blackman 2020). Extreme phenotypes in different cultivars of the same species have been bred: for example, shorter lifecycles to meet strict rotation requirements, or highly delayed bolting to increase storage organ size (for classic Brassica examples, see Cheng et al. 2016; Helal et al. 2016).

**Figure 2.**
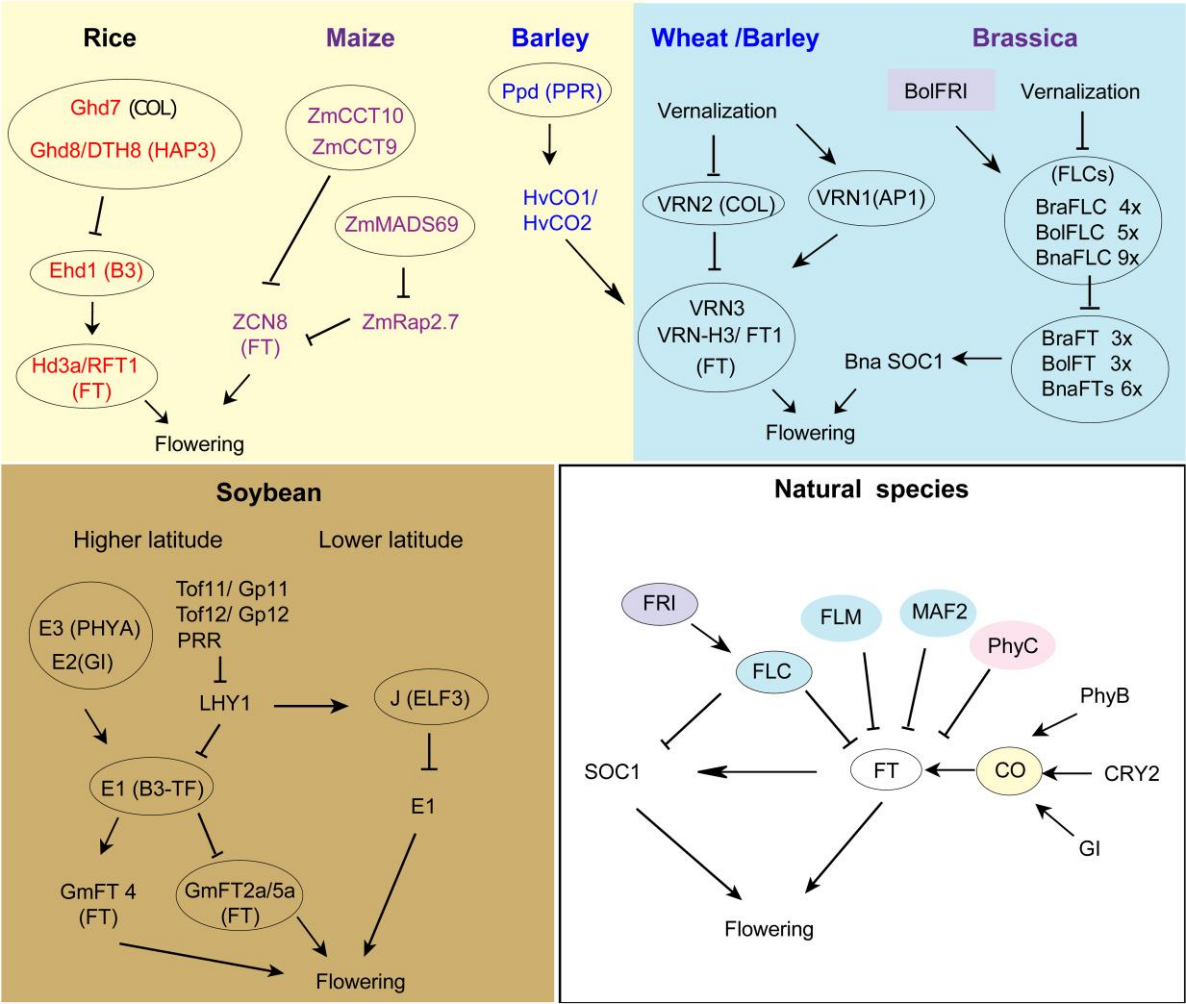
Natural variation in flowering pathways, comparing domesticated crops and natural species. Nodes circled in black in each pathway represent the major nodes with high allelic diversity. Yellow box shows selected photoperiodic regulators in rice (red text), maize (purple text), and barley (blue text). Extensive natural variation also occurs in vernalization regulators in barley (blue box), wheat, and brassica crops. Soybean breeding has predominantly targeted circadian clock components (pale brown box). White box: loci showing natural variation in *A. thaliana*.

Wild rice and maize (*Zea mays*) species are naturally found in low latitudes and exhibit characteristics of short-day plants (Matsuoka et al. 2002; Huang et al. 2012). To adapt to cultivation regions in high latitudes with longer daylight periods, the selection process involved the frequent choice of loss-of-function or weakened alleles of long-day suppressor genes enabling domesticated cultivars to flower early and maximize yield (Izawa 2007; Zhang et al. 2023b). For example, QTL mapping has revealed natural mutations in *Ghd7* and *Ghd8*/*DTH8* with reduced functions that enable rice to be cultivated in temperate regions. Ghd7 is a CCT domain protein showing homology to *Arabidopsis* CO and CO-LIKE (COL) (Xue et al. 2008), while *Ghd8*/*DTH8* encodes a HAP3 subunit of a CCAAT-box binding protein the HEME ACTIVATOR PROTEIN (HAP) complex (Fig. 2) (Wei et al. 2010; Yan et al. 2011; Dai et al. 2012). In *A. thaliana*, CO and HAP also form a transcriptional activation complex to modulate *FT* expression (Wenkel et al. 2006; Gnesutta et al. 2017; Lv et al. 2021). Similarly, 2 COL genes, *ZmCCT9* and *ZmCCT10*, have been cloned as flowering-time QTLs in maize (Yang et al. 2013; Huang et al. 2018). Extensive studies on natural variation in rice has identified *Hd16* and *Hd6*, encoding casein kinase I (CKI) and alpha subunit of casein kinase II (CKII alpha), respectively (Takahashi et al. 2001; Hori et al. 2013), and *DTH7*, which encodes a pseudo-response regulator protein whose expression is regulated by photoperiod (Fig. 2) (Liu et al. 2013; Gao et al. 2014).

In addition to disrupting LD suppressor genes, mutations that lead to enhanced flowering activators under both LD and SD have also been identified during domestication. For instance, the *FT* ortholog genes *Hd3a* and *RFT1* in rice and *ZCN8* in maize have been preferentially selected at different evolutionary times for local adaptation (Fig. 2) (Kojima et al. 2002; Komiya et al. 2008; Ogiso-Tanaka et al. 2013; Guo et al. 2018). Sequence polymorphisms in the regulatory and coding regions of *RFT1* and *ZCN8* may underlie divergence of flowering time among various cultivars and wild accessions (Fig. 2) (Ogiso-Tanaka et al. 2013; Guo et al. 2018). Moreover, rice *Ehd1*, which encodes a B-type response regulator that does not have a clear ortholog in *Arabidopsis*, promotes flowering under both LD and SD conditions (Doi et al. 2004). Finally, *ZmMADS69*, a MADS-box gene, contributed to the difference in flowering time between maize and its wild ancestor (teosinte) and may have played an important adaptive role during the expansion of maize from the tropics to temperate zones (Fig. 2) (Liang et al. 2019). ZmMADS69 likely downregulates the expression of the flowering time repressor *ZmRap2.7*, thereby alleviating the repression on *ZCN8* and promoting early flowering.

Unlike maize and rice, cultivated soybean has its origin near the temperate Yellow River region (Li et al. 2008). As such, the spread of soybean cultivars involved adaptation to both lower and higher latitudes. Evidence suggests that circadian clock genes were the primary targets for flowering time regulation during domestication (Lu et al. 2022a). Specifically, the adaptation of soybean to higher latitudes was facilitated by naturally occurring loss-of-function mutations in 5 flowering suppressors. Among them, *E1* encodes a legume-specific B3-like transcription factor acting as a suppressor in photoperiod pathway (Xia et al. 2012). E2 and E3 are likely the orthologs of Arabidopsis GI and PHYTOCHROME A (PHYA) (Watanabe et al. 2009; Watanabe et al. 2011). Tof11/Gp11 and Tof12/Gp12, paralogs of Arabidopsis PRR genes, function via homologs of LATE ELONGATED HYPOCOTYL1 (LHY1), a central component of the plant circadian clock. Tof11/Gp11 and Tof12/Gp12 promote *E1* expression and delay flowering under LD (Li et al. 2019; Gong 2020; Lu et al. 2020). The adaptation of soybean to lower latitudes, on the contrary, was driven by an impairment in *J*, the soybean ortholog of *Arabidopsis ELF3* that plays a highly conserved role maintaining circadian rhythms in different species (Fig. 2) (Lu et al. 2017). *J* promotes flowering under SD through repressing *E1*. Loss-of-function mutations in *J* led to an extended vegetative phase and higher yields at lower latitudes (Lu et al. 2017).

Barley (*Hordeum vulgare*) is primarily cultivated in temperate regions. The different varieties of barley can be categorized into 2 main groups based on their response to photoperiod: photoperiod-sensitive and photoperiod-insensitive. The *Ppd-1* gene, an *Arabidopsis PRR* ortholog, controls this photoperiod sensitivity (Turner et al. 2005). The presence of functional alleles of *Ppd-1* results in photoperiod insensitivity, allowing barley plants to flower under both LD and SD conditions (Fig. 2). As such, in higher latitudes with shorter summers, barley varieties with functional *Ppd-1* alleles are particularly advantageous as they enable earlier flowering and maturity. This trait ensures successful reproduction and higher yields in areas where the growing season is limited. By enabling barley to thrive in diverse environments, *Ppd-1* alleles contribute significantly to the cultivation of barley in temperate regions around the world.

Natural variations in vernalization are responsible for the differences in winter or spring growth habit observed in cereals (Kippes et al. 2018; Xu and Chong 2018). In wheat (*Triticum aestivum*), *VRN1* encodes a MADS-box transcription factor that shares homology with *Arabidopsis* AP1 (Yan et al. 2003; Konopatskaia et al. 2016). VRN2 (not the same protein as Arabidopsis VRN2!) and VRN3 show high similarities to *Arabidopsis* COL and FT, respectively (Yan et al. 2004, 2006). VRN2 functions as a floral repressor. Similar to *FLC* in *Arabidopsis*, the expression of *VRN2* is downregulated through vernalization. Loss-of-function alleles of *VRN2* lead to an increased level of *VRN1* and *VRN3*, thereby converting wheat from a winter annual to a spring annual growth habit (Fig. 2) (Yan et al. 2004). Dominant overexpressors of *VRN1* can also cause spring habit by overriding VRN2, and like *FLC*, *VRN1* alleles with differing expression influence both vernalization requirement and environmental sensitivity (Fu et al. 2005; Dixon et al. 2019). The core components of the vernalization pathway—VRN1, VRN2, and VRN3—are conserved between wheat and barley (Yan et al. 2004; Yan et al. 2006; Oliver et al. 2009). Notably, natural allelic variations in *VRN-H3* (*HvFT*) gene also contribute to difference in vernalization requirement in barley (Yan et al. 2006). Moreover, *EPS2*/*CEN*, which is a homolog of *Arabidopsis TFL1*, is involved in differentiating between winter and spring barley and has been selected and maintained during geographic range extension (Comadran et al. 2012).

Unlike the highly conserved flowering roles of the PEBP proteins, *CO* homologs and clock components, the *FLC* clade of MADS-box genes has not been reported as major flowering time regulators in crops outside of the Brassicaceae (Becker and Theißen 2003; Schilling et al. 2018). Members of other MADS-box clades quantitatively regulate phenology in crops: as well as the cereal *VRN2s* (AP1 family), *ODDSOC2* in barley is also a vernalization regulator but is cereal specific (Greenup et al. 2010). The *DORMANCY ASSOCIATED MADS-BOX* (*DAM*) genes involved in chilling requirement for bud break and bloom time across a range of perennial fruit trees (from apple to pear, peach, and sweet cherry) are most closely related to the SVP clade (Bielenberg et al. 2008; Falavigna et al. 2019; Calle et al. 2020), although an *FLC-like* gene has been found beneath QTL peaks in apple too, along with *AGL24* and *FT* homologs (Allard et al. 2016).

Compared with *Arabidopsis*, the “diploid” Brassica are mesohexaploids, and these diploids have further hybridized to form amphidiploids, which have preferentially retained copies of flowering time genes (Jones et al. 2018). *Brassica rapa* and *Brassica oleracea*, the main vegetable species, have at least 4 and 5 copies of *FLC* respectively, and their hybrid, *B. napus*, thus has at least 9, most of which have been implicated in flowering time variation between different cultivars and which underlie the majority of the main flowering time QTLs, with *FT* homologs accounting for several of the rest (for a comprehensive review, see Schiessl 2020; Song et al. 2020). Critically, these *FLC* copies have different expression sensitivity over cold as well as different expression dynamics between alleles, and it is total *FLC* expression, rather than expression of specific *FLC* paralogs, that best explains differences in cold requirement between cultivars (Schiessl 2020; Calderwood et al. 2021a). Given the very wide variation in flowering timing between conspecific Brassica crops, it is likely that the proliferation of *FLC*, each copy with different alleles, has contributed to the evolutionary space that permitted these different domesticated morphs (Calderwood et al. 2021a).

Taken together, while different alleles conferring accelerated or delayed flowering time were selected to aid the adaptation of crops to diverse cultivation areas at various latitudes, the underlying genes involved in these processes appear to be repeatedly selected across different crops. We direct the reader to further literature of major effect genes in other minor crop species (chickpea, Upadhyaya et al. 2015; sunflower, McAssey et al. 2016; apple, Urrestarazu et al. 2017; strawberry, Gaston et al. 2020; litchi, Lu et al. 2022b; flax, Saroha et al. 2022; pepper, Choi et al. 2023). Despite our focus on major crops, the conclusions are likely to extend to other crops. The main pattern observed is that during domestication, there is a notable preference for selection of master regulatory genes within each flowering time pathway—for example, orthologues of *CO* and *COL* in the photoperiod pathway, *FT* and *TFL1* in the florigen-related pathway, *PRR* and *ELF3* in the circadian clock, and *AP1* in the floral-promoting MADS-box gene family. Although the exact reasons for this are yet to be fully understood, variation at these genes may have provided compatibility with high-density field growth conditions and cultivation practices aimed at achieving high yield. In support of this notion, it has been shown that *Ghd7* has a broad impact on various traits in rice, including yield, plant height, and heading date (Xue et al. 2008). Similarly, in soybean domestication, Tof12/Gp12-dependent acceleration of maturity is associated with reduced dormancy and seed dispersal (Lu et al. 2020). Under this scenario, the aforementioned genes hold great potential as targets for crop breeding and future de novo domestication of wild crop-related species using genome editing approaches (Li et al. 2018; Zsogon et al. 2018; Yu et al. 2021).

In conclusion, a comparison between genes underpinning natural diversity in *Arabidopsis* and relatives compared with those selected during domestication of our major crops reveals crop domestication has utilized fewer loci with reduced allelic diversity. Perhaps nature hedges its bets maintaining high variation in the population, while crop domestication has prized predictability, at the cost of maximizing plant fitness in a fluctuating environment.

## Importance of *in natura* flowering analysis

A new realization in the field is the importance of undertaking experiments under field conditions; plants have not evolved to grow in the constant conditions we provide in the laboratory (Shimizu et al. 2011; Nishio et al. 2016; Hepworth et al. 2018; Zhao et al. 2021). So-called in natura field experiments are increasingly important for dissection of molecular mechanisms. Natural fluctuations in both photoperiod and temperature averages and ranges influence the plant transcriptome widely and have multifaceted effects on plant fitness (Nagano et al. 2019). Analysis of classic flowering time mutants revealed that under field conditions, many have much fewer phenotypic effects than in the laboratory (Wilczek et al. 2009; Song et al. 2018; Taylor et al. 2019). Conversely, field conditions may reveal critical mechanisms considered less important when studied in the laboratory (Brachi et al. 2010). Temperature dynamics in autumn, not winter, are likely to be the critical variable for vernalization (Duncan et al. 2015; Dixon et al. 2019; Hepworth et al. 2020) partly because as temperatures reduce during autumn their fluctuations have different effects on *FLC* silencing depending on their precise range and timing (Antoniou-Kourounioti et al. 2018; Hepworth et al. 2018). Early freezing is one such effect (Zhao et al. 2021). Temperature fluctuations also turn out to be key to the function of the photoperiod pathway in the field, reducing the impact of mutations in *FKF1* and *GI* compared with the laboratory but revealing important roles for *ELF3* and *PHYA* in natural conditions (Song et al. 2018; Kinmonth-Schultz et al. 2023). By investigating the expression dynamics of haplotypes in the field and the subsequent fitness of plants carrying these haplotypes, avoidance of precocious flowering in autumn rather than in spring was revealed as a key driver for the vernalization pathway in a sub-artic environment, with high-expression “slow vernalizing” alleles providing protection against precocious flowering (Hepworth et al. 2020). This fits with findings by Fournier-Level et al. (2022) that late-flowering alleles are promoted in environments with high seasonal temperature fluctuation. Most of these studies monitored the behavior of genes in the field itself: however, many then recapitulated these observations in laboratory settings, in order to test and quantify the observed environmental drivers of different molecular responses. This combination of approaches nullifies some of the distorting effects of laboratory investigation, while exploiting its power to verify interactions, and is likely to be of continuing importance to future research on plant environmental sensitivity.

## Analyzing flowering in a new species

What lessons have emerged from all these studies that will influence identification of critical nodes for flowering regulation in newly researched species? Research on rice, wheat, barley, and soybean shows that many of the same principles hold, and *A. thaliana* has (and continues to have) lessons for the functioning of fundamental pathways. A favored strategy in crops with the advent of deep sequencing has been to use RNA-seq analysis and GWAS to look for gene expression variation and linked polymorphism. In the original GWAS studies in *A. thaliana*, *FRI* and *FLC* were not detected as significantly associated loci partly due to population structure in the original set of 96 lines (Atwell et al. 2010), although larger studies with more advanced statistical tools have improved on this (Sasaki et al. 2015). Subsequent QTL (Shindo et al. 2005) and molecular analysis (Li et al. 2014) showed that allelic heterogeneity (different haplotypes having the same phenotype) also reduces the statistical significance, so preventing detection by GWAS. Sample size will also influence detection—GWAS and QTL studies require very large sample sizes, and they can only map those differences that are captured between the initial parental strains. The environmental sensitivity of flowering time is also probably one of the largest difficulties in any single GWAS or QTL experiment. Field studies have demonstrated that much selection is conditional—and the power of GWAS studies to investigate GxE interactions can be low (Sasaki et al. 2015).

So going forward, this is we learned that will be useful to define flowering time regulators in a new species, enabling us to breed staple crops adapted to climate change, or develop underutilized crops:

1. Make the most of the considerable molecular knowledge of flowering time gene action.
2. Select GWAS on diversity panels (Harper et al. 2012; 1001 Genomes Consortium 2016) to detect a wide variety of natural alleles affecting flowering; but use intercrossed mapping populations to better identify loci of critical nodes such as FLC (Brachi et al. 2010; Song et al. 2020).
3. Once a critical quantitative node has been identified, explore the dynamics of standing variation of that gene across a wide diversity of accessions. SNPs, presence/absence, transposons etc. within haplotypes will assist in generating targeted, quantitative change in flowering responses while reducing interference with pleiotropic functions of these master regulators (Turner et al. 2005; Liu et al. 2014; Bao et al. 2019; Dixon et al. 2019; Song et al. 2020).
4. Dynamic genes require dynamic methods: many of the critical nodes change quantitatively over time, so time-course analyses are essential (Shindo et al. 2006; Duncan et al. 2015; Nagano et al. 2019; Schiessl et al. 2019; Calderwood et al. 2021a), and tools are becoming available for easier comparison of transcriptomics (Calderwood et al. 2021b).
5. Move molecular experimentation into the field early in the research pipeline—but then return to the laboratory to validate and quantify the results.

Knowledge from molecular research has had high barriers to implementation in crops, in part because phenotypic analysis from the laboratory does not always translate into the field (Atwell et al. 2010). One reason for this is lack of knowledge about critical field conditions, such as the temperature profiles required for vernalization (Hepworth et al. 2018; Dixon et al. 2019) or the light patterns that induce *FT in natura* (Song et al. 2018). However, by combining field studies with more accurate simulation in the laboratory, *Arabidopsis* research is developing a range of new methods for understanding, and crucially predicting, how pathways respond and control flowering in the changing field conditions (Antoniou-Kourounioti et al. 2018; Song et al. 2018; Nagano et al. 2019). With climate change challenging the key mechanisms plants rely on for their timing, *Arabidopsis* research remains critical to fundamental knowledge and plant breeding alike.

Mechanistic information from *A. thaliana* has significantly accelerated understanding of flowering time regulation in all plant species. This is readily recognized by breeding companies (Enza Zaden Box 3). Thus, for the timely production of a range of climate-proof crops, we need to focus on expanding our mechanistic understanding of flowering time gene function in natural environments and those mechanisms that have changed during adaptation. This will be the fastest route to open new opportunities for crop improvement.

Box 3. **Impact of flowering time research to advance plant breeding.**

Drs. Xana Verweij and Jeroen Rouppe van der Voort Global Biotech director and Research Manager IP/External Projects at Enza Zaden Research and Development.

Flowering time plays a crucial role in breeding. There are numerous examples ranging from breeding for different seasonal product types in e.g. cauliflower and lettuce (Leijten et al. 2018), balancing the switches from vegetative to generative plant growth in peppers, climate zone adaptation in onion types (Lee et al. 2013a, 2013b), to escaping Phytophthora disease pressure in potato (by planting “early varieties”) and seed quality and yield (applicable to any seeded crop variety). In addition, the finding that flowering time regulators are involved in many other plant developmental pathways stresses the importance of obtaining a deep understanding of flowering-related processes in food crops. The advent of the application of omics tools to create any type of data, and the increased capabilities for analyzing such data sets finally enables breeders to identify the key loci and allelic variation to breed for. The work done by fundamental research is essential to uncover the spatio-temporal regulation of flowering time laying the foundations of knowledge that can be translated by breeding companies to create predictable and adaptable crop products. We aim to identify floral pathway integrators in different crops which might serve as breeding targets, allowing us to design strategies towards optimal flowering and robust fruit and seed production even under adverse growing conditions. Therefore, it is essential to bring fundamental research and commercial crop breeding objectives closer together. This will guarantee that we work in synergy for the relevant traits that can have a positive impact in our agricultural systems by e.g. developing resilient crops with optimal yield and quality potential and minimal trade-off effects.

**Outstanding questions**

- To what extent have we identified all the pathways regulating flowering time in distinct climates?
- Do the same principles hold broadly across natural plant species and most crops?
- What makes some floral regulators such sensitive targets to general transcriptional/epigenetic regulators?
- How much does noncoding transcription/long noncoding RNA contribute to altering regulatory dynamics in fluctuating natural environments?

## Data Availability

Sequencing data presented in this article is available on the Short Read Archive (SRA) under the project reference PRJNA1088482.

## Dive Curated Terms

The following phenotypic, genotypic, and functional terms are of significance to the work described in this paper:

NTL8 Gramene: AT2G27300
NTL8 Araport: AT2G27300

SPL15 Gramene: AT3G57920
SPL15 Araport: AT3G57920

FKF1 Gramene: AT1G68050
FKF1 Araport: AT1G68050

PHYA Gramene: AT1G09570
PHYA Araport: AT1G09570

FLK Gramene: AT3G04610
FLK Araport: AT3G04610

FPA Gramene: AT2G43410
FPA Araport: AT2G43410

PIF4 Gramene: AT2G43010
PIF4 Araport: AT2G43010

PIF7 Gramene: AT5G61270
PIF7 Araport: AT5G61270

SPA1 Gramene: AT2G46340
SPA1 Araport: AT2G46340

SVP Gramene: AT2G22540
SVP Araport: AT2G22540

AP2 Gramene: AT4G36920
AP2 Araport: AT4G36920

MIR172a Gramene: AT2G28056
MIR172a Araport: AT2G28056

FLC Gramene: AT5G10140
FLC Araport: AT5G10140

FTIP1 Gramene: AT5G06850
FTIP1 Araport: AT5G06850

h3k27me3 CHEBI: CHEBI:85045

FLM Gramene: AT1G77080
FLM Araport: AT1G77080

COLDAIR Gramene: COLDAIR
COLDAIR Araport: COLDAIR

FT Gramene: AT1G65480
FT Araport: AT1G65480

SOC1 Gramene: AT2G45660
SOC1 Araport: AT2G45660

AP1 Gramene: AT1G69120
AP1 Araport: AT1G69120

FUL Gramene: AT5G60910
FUL Araport: AT5G60910

CAL Gramene: AT1G26310
CAL Araport: AT1G26310

LFY Gramene: AT5G61850
LFY Araport: AT5G61850

CO Gramene: AT5G15840
CO Araport: AT5G15840

FRI Gramene: AT4G00650
FRI Araport: AT4G00650

ZTL Gramene: AT5G57360
ZTL Araport: AT5G57360

COP1 Gramene: AT2G32950
COP1 Araport: AT2G32950

GI Gramene: AT1G22770
GI Araport: AT1G22770

ELF3 Gramene: AT2G25930
ELF3 Araport: AT2G25930

FD Gramene: AT4G35900
FD Araport: AT4G35900

TFL1 Gramene: AT5G03840
TFL1 Araport: AT5G03840

QKY Gramene: AT5G03840
QKY Araport: AT5G03840

SYP121 Gramene: AT3G11820
SYP121 Araport: AT3G11820

PFT1 Gramene: AT1G25540
PFT1 Araport: AT1G25540

MIR156c Gramene: AT4G31877
MIR156c Araport: AT4G31877

VIN3 Gramene: AT5G57380
VIN3 Araport: AT5G57380

VRN5 Gramene: AT3G24440
VRN5 Araport: AT3G24440

TSF Gramene: AT4G20370
TSF Araport: AT4G20370

MFT Gramene: AT1G18100
MFT Araport: AT1G18100

ELF7 Gramene: AT1G79730
ELF7 Araport: AT1G79730

CBP20 Gramene: AT5G44200
CBP20 Araport: AT5G44200

CBP80 Gramene: AT2G13540
CBP80 Araport: AT2G13540

PRP8 Gramene: AT1G80070
PRP8 Araport: AT1G80070

FCA Gramene: AT4G16280
FCA Araport: AT4G16280

FY Gramene: AT5G13480
FY Araport: AT5G13480

CstF64 Gramene: AT1G71800
CstF64 Araport: AT1G71800

CstF77 Gramene: AT1G17760
CstF77 Araport: AT1G17760

APRF1 Gramene: AT5G14530
APRF1 Araport: AT5G14530

TOPP4 Gramene: AT2G39840
TOPP4 Araport: AT2G39840

PHYB Gramene: AT2G18790
PHYB Araport: AT2G18790

PHYC Gramene: AT5G35840
PHYC Araport: AT5G35840

MAF2 Gramene: AT5G65050
MAF2 Araport: AT5G65050

GIS5 Gramene: AT5G63960
GIS5 Araport: AT5G63960

AT5G63960 Gramene: AT5G63960
AT5G63960 Araport: AT5G63960
